# Embroidered Silk Fibroin Scaffolds for ACL Tissue Engineering

**DOI:** 10.3390/ijms27010137

**Published:** 2025-12-22

**Authors:** Yasir Majeed, Clemens Gögele, Cindy Elschner, Christian Werner, Tobias Braun, Judith Hahn, Ricardo Bernhardt, Udo Krause, Bernd Minnich, Gundula Schulze-Tanzil

**Affiliations:** 1 Institute of Anatomy and Cell Biology, Paracelsus Medical University, Prof.-Ernst-Nathan Straße 1, 90419 Nuremberg, Germany; yasir.majeed@pmu.ac.at (Y.M.); clemens.goegele@pmu.ac.at (C.G.); christian.werner@pmu.ac.at (C.W.); tobias.braun@pmu.ac.at (T.B.); 2 Division Polymer Materials Engineering, Leibniz Institute of Polymer Research Dresden (IPF), Hohe Straße 6, 01069 Dresden, Germany; elschner@ipfdd.de (C.E.); bernhardt@ipfdd.de (R.B.); 3 Department Science Support & International Collaborations, Faculty of Medicine and University Hospital Carl Gustav Carus, TUD Dresden University of Technology, Fetscherstraße 74, 01307 Dresden, Germany; judith.hahn@ukdd.de; 4 Seidenkokon Native Silk GmbH, Hauptstraße 11a, 01920 Nebelschütz, Germany; udo.krause@seidenkokon.de; 5 Department of Environment & Biodiversity, Paris Lodron University Salzburg, 5020 Salzburg, Austria; bernd.minnich@plus.ac.at

**Keywords:** anterior cruciate ligament, ligament fibroblasts, silk, P(LA-CL), embroidery

## Abstract

Anterior cruciate ligament (ACL) rupture causes joint instability and increases the risk of osteoarthritis due to the ligament’s limited healing capacity. Silk, particularly from *Bombyx mori*, combines high cytocompatibility with robust biomechanical properties. Its main components are fibroin and sericin, with the latter usually being removed to reduce immunogenicity and improve biocompatibility. Silk threads were processed either as raw silk (designated as “untreated”) or subjected to a patented degumming procedure (DE102021118652A1) to obtain purified silk. Both variants were used alone or in combination with poly(L-lactic acid-co-caprolactone) (P(LA-CL)) fibers, yielding four scaffold groups: untreated silk, purified silk, untreated silk/P(LA-CL), and purified silk/P(LA-CL). Three-layer scaffolds were fabricated using a zigzag embroidery pattern. Structural analysis revealed scaffold porosity of ≈38% for silk, ≈46% for purified silk, and up to ≈70% for scaffolds containing P(LA-CL). Uniaxial tensile testing showed that purified silk scaffolds achieved the highest maximum force at break (≈684 N), whereas elongation at maximum force was limited in the hybrid scaffolds—silk/P(LA-CL) ≈ 28% and p-silk/P(LA-CL) ≈ 32%—despite the high intrinsic extensibility of P(LA-CL). All scaffolds supported cell adhesion and showed no cytotoxicity. P-silk and p-silk/P(LA-CL) scaffolds exhibited the highest fibroblast adherence and pronounced paxillin expression, indicating strong cell–material interactions. Gene expression of ligament-related ECM components and connexin 43 was maintained across all groups. These results demonstrate that embroidered silk fibroin scaffolds provide a reproducible architecture with tunable porosity and mechanical properties, supporting fibroblast colonization and ligament-specific ECM expression. Such scaffolds represent promising candidates for ACL tissue engineering and future graft development.

## 1. Introduction

Anterior cruciate ligament (ACL) is the most commonly injured ligament in the knee [1], and there is a constant rise in the incidence as well [2]. ACL repair is usually essential, as the majority of ACL injuries do not heal and increase the risk of both joint instability and early-onset osteoarthritis. The current practice involves surgical repair using auto- or allografts from the hamstring or patellar tendons [2]. However, the procedure carries some limitations, such as the limited availability of the auto- and allografts [3] and donor site morbidities. This has stirred a rising interest to look for tissue-engineering-based alternatives as potential ACL grafts [3,4,5]. It is essential that the graft should be biocompatible and have the biomechanical strength comparable to the native ACL, since the knee joint is an area of high biomechanical loading [6]. Both natural biomaterials (silk) and synthetic ones (poly-L-lactic acid (PLLA)) have been explored in the literature as potential candidates for preparing ACL [7,8]. Silk has been in medical use for a long time, notably as silk sutures [9], since it offers properties such as hierarchical organization, structural stability, and slow degradation both in vitro [10] and in vivo [11]. Additionally, its biomechanical properties closely resemble those of the native ACL [12]. Silk worm (*Bombyx [B.] mori*)-derived silk consists of two components: fibroin and sericin [13,14]. Since sericin is immunogenic, it is removed to make silk suitable for medical use. Moreover, its removal also enhances silk’s biocompatibility and processability, which are important for textile engineering applications. Conventional sericin removal procedures may adversely affect silk biocompatibility due to the use of harsh chemicals and alterations in pH [15]. A novel, less aggressive procedure was patented recently (DE102021118652A1). This approach involves washing silk in two consecutive heated (95 °C) water baths using electrolytic water, with the second bath supported by ultrasound sonication, followed by steam and infrared ray treatment. Finally, ultraviolet radiation (UV) is applied to sterilize the fibers before the threads are dried. Silk has previously been used for ACL tissue engineering, and braided scaffolds made of synthetic polymers or silk have shown promising mechanocompetence [16]. However, we are studying an alternative scaffold fabrication method: machine embroidery technology. Embroidery helps us to generate scaffolds with a three-dimensional (3D) structure and biomechanical characteristics that match the dimensions and properties of the native rabbit ACL, since the rabbit could serve as a future animal model for in vivo testing [4]. The embroidery pattern is customizable, allowing for the freedom and flexibility of design. This customization is achieved by varying stitch lengths, angles, and duplication shifts [4]. Based on the hypothesis that degummed silk fibroin multifilaments can be used to embroider ACL scaffolds with a biomimetic design, this study explores the cytocompatibility and behavior of ACL fibroblasts on embroidered silk-fibroin-based scaffolds for ACL tissue engineering.

To the best of our knowledge, this is the first project in the currently available literature to study the impact of embroidered silk scaffolds on facilitating ACL tissue engineering.

## 2. Results

### 2.1. Characteristics of Silk and Silk-Based Scaffolds

The machine embroidery technique was successfully used to process natural silk fibers of both purification grades, as well as the resorbable monofilament suture material P(LA-CL). All individual materials and combinations thereof were suitable for the embroidery process without causing fiber breakage. The absorbable monofilament made of P(LA-CL) could be reliably integrated with silk fibers to form stable scaffold structures, demonstrating the feasibility of combining different biomaterials within a single embroidery-based manufacturing process.

#### 2.1.1. Silk Fiber Dimensions

**Figure 1:**
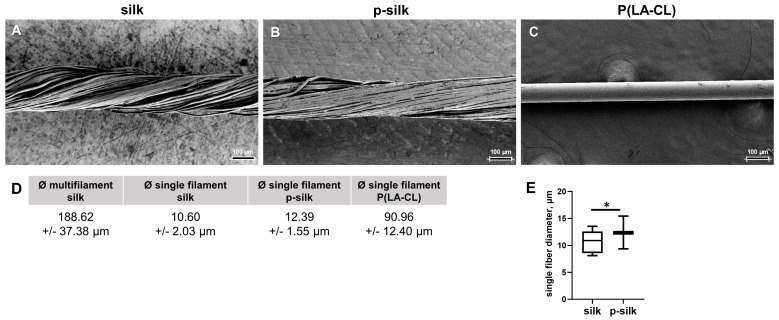
(**A**–**E**) SEM images of the yarn materials used in the study. (**A**) Untreated silk, (**B**) purified (p-) silk, (**C**) poly-L-lactic acid-co-caprolactone (P(LA-CL)). (**D**) The mean fiber diameters and standard deviation are listed, measured in DAPI stained scaffolds. (**E**) The single-fiber diameter of p-silk was significantly higher than untreated silk, * *p* < 0.05. Scale bars: 100 µm.

Figure 1A–C highlights the pronounced differences in filament cohesion and surface morphology among the examined materials. The untreated silk yarn (silk) consists of loosely twisted filaments with minimal cohesion, which can be readily separated, reflecting the unprocessed state of the material (Figure 1A). In contrast, purified silk (p-silk) exhibited a more compact structure with tightly adhering filaments, suggesting enhanced intermolecular interactions resulting from the degumming process (Figure 1B). The resorbable monofilament suture (P(LA-CL)), shown on the far right, exhibits a smooth and homogeneous polymer surface, characteristic of synthetic degradable materials and fundamentally distinct from the filamentous architecture of silk (Figure 1C).

The fiber dimensions were measured in the embroidered DAPI-stained scaffolds (Supplemental Figure S1). Silk fibers were typical multifilaments consisting of about 180 single filaments, with an overall diameter of 188.62 ± 37.38 μm (Figure 1D). The diameter of the single filaments for untreated silk, purified silk, and P(LA-CL) fibers in the scaffold variants was 10.60 ± 2.03 μm, 12.39 ± 1.55 μm, and 90.96 ± 12.4 μm, respectively (Figure 1D). Purified silk had a significantly larger fiber diameter compared to the untreated silk (Figure 1E).

#### 2.1.2. Characteristics of Textile Scaffold Structure

**Figure 2:**
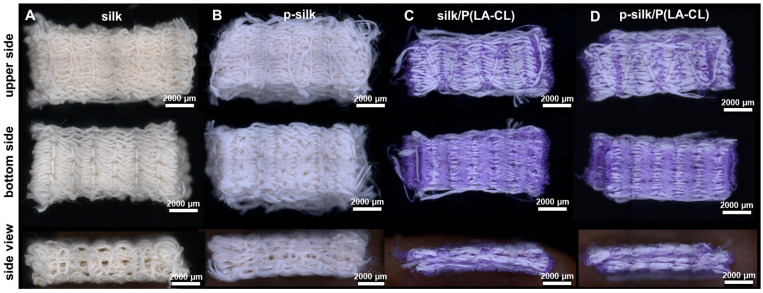
(**A**–**D**) Embroidered silk scaffold types. (**A**) untreated silk, (**B**) purified (p-) silk, (**C**) untreated silk/P(LA-CL), (**D**) purified silk/P(LA-CL). P(LA-CL): poly L-lactic acid-co-caprolactone.

All embroidered constructs (see Section 4.2) consisted of three layers, yielding very dense and mechanically robust textile structures. Visual inspection revealed that scaffolds composed entirely of silk could be clearly distinguished from those made of a blend of silk and purple-dyed absorbable suture material. Due to the greater diameter of silk multifilaments compared to that of resorbable material (Figure 2), silk scaffolds possess a more compact structure in contrast to the hybrid scaffolds consisting of silk and P(LA-CL) scaffolds.

**Figure 3:**
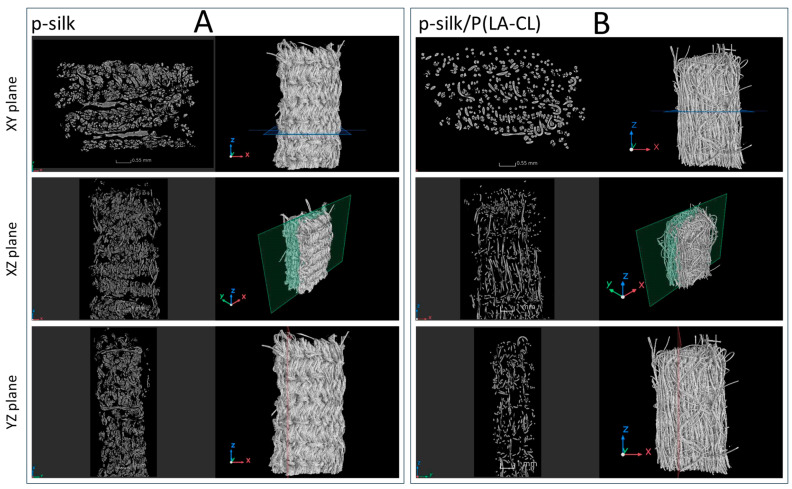
(**A**,**B**) 3D reconstructions and slice images of all three cross-sectional planes, exemplified for the groups p-silk and p-silk/P(LA-CL). The positions from which the slice images were generated are marked in each case. (**A**): p-silk: purified silk; (**B**): P(LA-CL): poly L-lactic acid-co-caprolactone.

The disparity in packing density of the scaffolds is discernible in the cross-sectional images of the µCT scans (cf. Figure 3).

**Figure 4:**
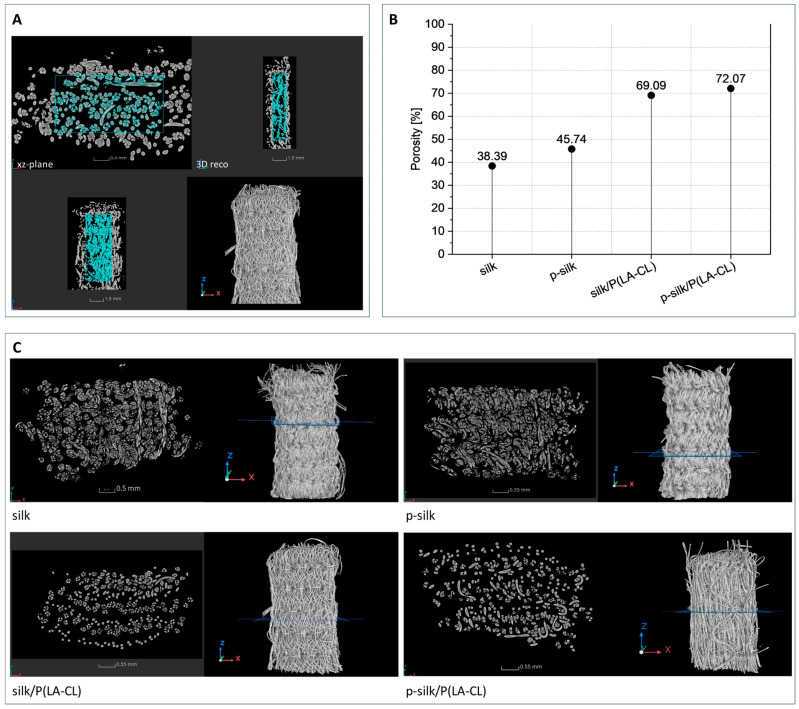
(**A**–**C**) Porosity determination based on a volume located in the scaffold. The proportions of fibers and voids were determined from the total volume of this three-dimensional region of interest, and the total porosities were calculated from this (**A**). Porosity of the scaffold types, determined from one representative sample (**B**). Comparison of exemplary selected x–y cross-sectional images from all sample groups (**C**). p-silk: purified silk; P(LA-CL): poly-L-lactic acid-co-caprolactone.

This particular type of textile scaffolds is characterized by a multitude of interstices, cavities between individual fibers in the silk multifilaments, pores that emerge from the interlacing of upper and lower threads, and cavities that are a consequence of the three layers of the scaffold lying on top of each other. The resulting cavity system is interconnected, and single interstices are not enclosed on all sides, as is the case, for example, with porous ceramic materials or scaffolds produced by 3D filament printing (Figure 4).

Scaffolds fabricated from untreated silk exhibited the densest textile architecture and, correspondingly, the lowest porosity, as illustrated in Figure 4. In contrast, scaffolds fabricated from purified silk showed a moderately higher porosity (≈46%) compared to those from untreated silk (≈38%). As these values are based on single specimens per silk type, the observed difference cannot be considered statistically significant. Using monofilament suture material for the lower thread clearly increases the porosity of the scaffolds to 69% for silk/P(LA-CL) and 72% for p-silk/P(LA-CL). The smaller diameter of the monofilament—about half that of the silk yarn—explains the differences observed, even though the embroidery pattern itself remains unchanged. Because synthetic monofilament is stiffer than multifilament silk yarn, it pulls the silk fibers more tightly together, resulting in a denser structure. This increased compactness is readily apparent in the µCT cross-sectional images (see Figure 3).

#### 2.1.3. Biomechanical Characteristics of Fiber Materials and Scaffolds

**Figure 5:**
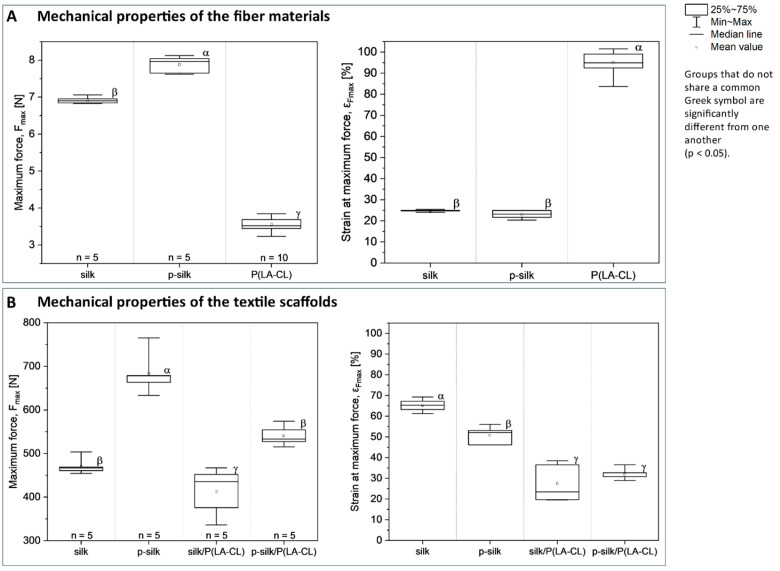
(**A**,**B**) Mechanical properties of fibers and embroidered silk-based scaffolds determined by uniaxial tensile testing. Boxplots display the maximum force and strain at maximum force. The boxes represent the interquartile range (25th–75th percentile), the whiskers indicate the minimum and maximum values, the horizontal lines show the medians, and the circles denote the mean values. Distinct Greek symbols indicate statistically significant differences between groups (*p* < 0.05), while groups sharing the same letter are not significantly different. The figure compares the results of the uniaxial tensile test for the thread material (**A**) and the textile scaffold variants (**B**). p-silk: purified silk, P(LA-CL): poly-L-lactic acid-co-caprolactone.

The mechanical properties of the embroidered silk-based scaffolds were evaluated by uniaxial tensile testing. Maximum force (N) and elongation at maximum force (%) were compared across scaffolds with different yarn compositions. Boxplots depict the distribution of the data, and statistically significant differences between groups are indicated by distinct Greek symbols (*p* < 0.05) (Figure 5).

A comparative analysis of the raw materials revealed that silk threads exhibited a breaking strength approximately double that of the synthetic sutures. Furthermore, the removal of sericin from untreated silk resulted in a significant increase in its load-bearing capacity. In contrast, strain at failure was consistent between the silk variants, averaging 25% for untreated silk and 23% for p-silk. This is in contrast to the monofilament resorbable suture, which displayed a substantially higher mean strain of 95%.

The mechanical performance of the embroidered scaffolds was governed by both yarn selection and textile structure. Scaffolds fabricated with purified silk for both the upper and lower threads achieved the highest ultimate tensile forces, with the p-silk configuration significantly surpassing all other groups at 684 N. Overall, the use of P(LA-CL) as the lower thread was associated with a decrease in tensile performance. Accordingly, scaffolds featuring a hybrid design of untreated silk and P(LA-CL) threads demonstrated the lowest failure threshold, registering a value of 413 N.

Although the synthetic suture material displays a much higher intrinsic elongation (≈95%), scaffolds containing P(LA-CL) as the lower thread show markedly lower strain at maximum force. Quantitatively, strain at maximum force was 65.4% (silk), 50.7% (p-silk), 27.6% (silk/P(LA-CL)), and 32.0% (p-silk/P(LA-CL)). Purification (sericin removal) reduced mean strain by 14.7 percentage points (65.4 → 50.7; ≈22.5% relative decrease). The presence of P(LA-CL) as lower thread reduced strain much more strongly: silk → silk/P(LA-CL) fell by 37.8 percentage points (≈57.8% relative reduction), and p-silk → p-silk/P(LA-CL) fell by 18.7 percentage points (≈36.9% relative reduction).

### 2.2. No Cytotoxic Effects of the Scaffolds on LCL Fibroblasts

**Figure 6:**
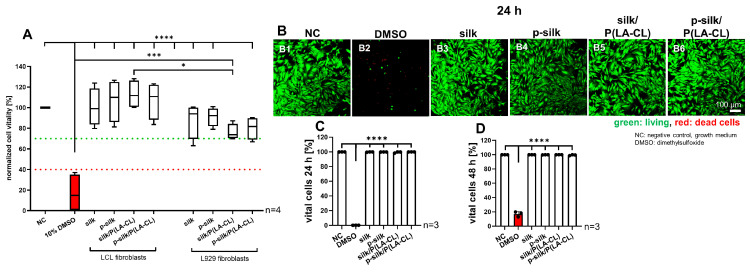
(**A**–**D**) Cytotoxicity assay with lapine cruciate ligament (LCL) and L929 fibroblasts exposed 24 h to scaffold extracts (**A**) and LCL fibroblast vitality after 24 h (**B**–**D**). (**A**) Green line: 70%: threshold to low cytotoxicity; red line: 40%: threshold to high cytotoxicity. * *p* < 0.05. ANOVA multiple comparison (Tukey post hoc test) was performed to detect significant differences between the groups. *n* = 4. (**B1**–**B6**) Life/death assay with LCL fibroblasts after 24 h of incubation with extracts. Scale bar: 100 µm, 100× magnification. (**C**) Calculation of % surviving cells after 24 h. *n* = 3. (**D**) Calculation of % surviving cells after 48 h. *n* = 3. NC: negative control (growth medium, (**B1**)); DMSO: dimethylsulfoxide (cytotoxic control, (**B2**)). (**B3**): silk, p-silk: purified silk (**B4**). P(LA-CL): poly-L-lactic acid-co-caprolactone combined with silk (**B5**) or with purified silk (**B6**). one-way ANOVA multiple comparison and 1% Rout outlier test were used. * *p* < 0.05, *** *p* < 0.001, **** *p* < 0.0001.

It is essential to assess whether the scaffold material is compatible for the growth of the fibroblasts. Therefore, a cytotoxicity assay was performed using the scaffold extracts (after incubating them for 48 h in growth medium) adding them to monolayer cultured LCL and L929 fibroblasts. Both fibroblast types were exposed to the scaffold extracts for 24 h, and then cytotoxicity was assessed. The assay revealed that the scaffold material was not cytotoxic to both cell types, and the cell vitality was significantly higher in all the scaffold groups compared to the cells treated with 10% DMSO (Figure 6A). All fibroblasts exposed to scaffold extracts showed a vitality higher than the threshold of 70%. The LCL fibroblasts exhibited generally higher vitality compared to the L929 fibroblasts exposed to the same scaffold extracts. This difference between both cell types reached the significance level regarding the purified silk/P(LA-CL) group. High levels of cell viability could also be visualized by a life/death stain of LCL fibroblasts exposed for 24 or 48 h to scaffold extracts with no significant difference compared to cells incubated with growth medium. There were only a few dead cells detectable (Figure 6B–D).

**Figure 7:**
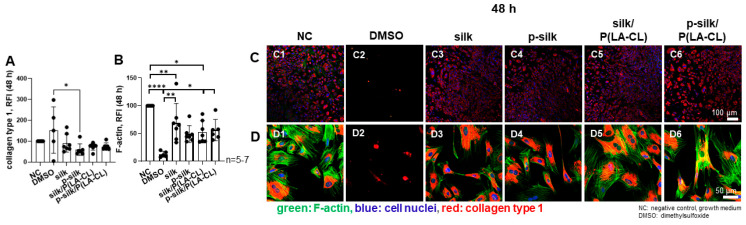
(**A**–**D**) Collagen type 1 expression/F-actin organization after 48 h exposition to scaffold extracts (**C1**–**C6**,**D1**–**D6**). (**A**) Relative fluorescence intensity (RFI) of collagen type. (**B**) RFI of F-actin. *n* = 5–7. Collagen type 1 expression (red)/F-actin organization (green) at lower ((**C1**–**C6**), scale bar: 100 µm, 100× magnification) and higher magnification ((**D1**–**D6**), scale bar: 50 µm, 400× magnification) after exposing LCL fibroblasts for 48 h with scaffold extracts. *n* = 5–7. NC: negative control (growth medium, (**C1**,**D1**)). DMSO: dimethylsulfoxide (cytotoxic control, (**C2**,**D2**)). (**C3**,**D3**): silk, p-silk: purified silk (**C4**,**D4**). P(LA-CL): poly-L-lactic acid-co-caprolactone combined with silk (**C5**,**D5**) or with purified silk (**C6**,**D6**). One-way ANOVA multiple comparison and 1% Rout outlier test were used. Collagen type 1 and F-actin expression was normalized to DAPI stained cell nuclei. * *p* < 0.05, ** *p* < 0.01, **** *p* < 0.0001.

Collagen type 1 expression and cell morphology was not significantly affected by exposing the LCL fibroblasts to scaffold extracts compared to culturing in growth medium (control cultures). Cells treated with DMSO showed variable collagen expression per cell. The organization of the intracellular F-actin network did not change in cells treated with extracts compared to those cultured with growth medium (negative control: NC). However, the intensity of F-actin staining decreased significantly in the cells treated with scaffold extracts compared to the control (NC, Figure 7). Except for p-silk, the F-actin expression was significantly higher in extract-treated monolayer cells compared to those cultured with DMSO (10%). In contrast, DMSO caused severe cell disintegration and morphological changes. Collagen type 1 expression was detectable, but F-actin networks could be barely demonstrated in DMSO-treated cells.

### 2.3. LCL Fibroblasts Survive on Silk Scaffolds

**Figure 8:**
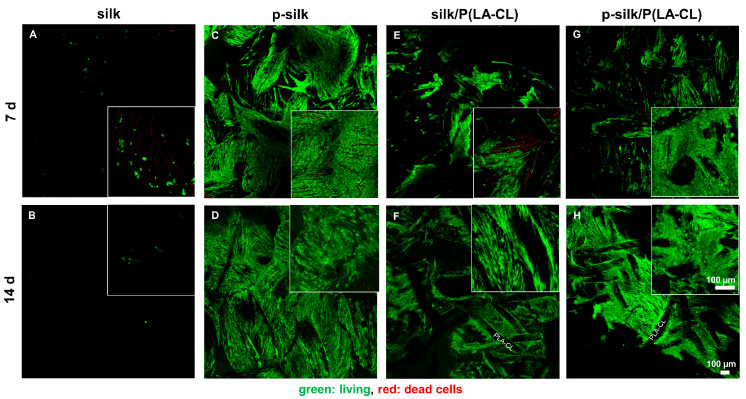
(**A**–**H**) Results of life/death assay of colonized scaffolds. The inset depicts a higher magnification. The majority of LCL fibroblasts are living (green), and only few dead cells (red) are detectable. (**A**,**B**) untreated silk, (**C**,**D**) purified (p-)silk, (**E**,**F**) untreated silk/P(LA-CL), (**G**,**H**) purified silk/P(LA-CL). P(LA-CL): poly-L-lactic acid-co-caprolactone. (**A**,**C**,**E**,**G**) 7 d. (**B**,**D**,**F**,**H**) 14 d. Scalebar: 100 µm; 100× magnification. Insets: 200× magnification.

A viability (life/death) assay was performed to visualize the living and dead cells on the scaffolds, both at day 7 and 14 using Confocal Laser Scanning Microscope (CLSM). Untreated silk scaffolds exhibited the least cell adherence of living cells (Figure 8A,B), whereas p-silk showed the highest (Figure 8C,D). Accordingly, cells appeared more rounded on untreated silk. Compared to untreated silk, the addition of P(LA-CL) to untreated silk enhanced the adherence of living cells (Figure 8E,F). Cells showed an elongated shape following the filaments. A similar picture of a dense cell layer adhering to scaffold fibers became evident for cells cultured on purified silk/P(LA-CL) (Figure 8G,H).

### 2.4. Expression of Cytoskeletal Components and Collagen Type I on Silk Scaffold Variants

**Figure 9:**
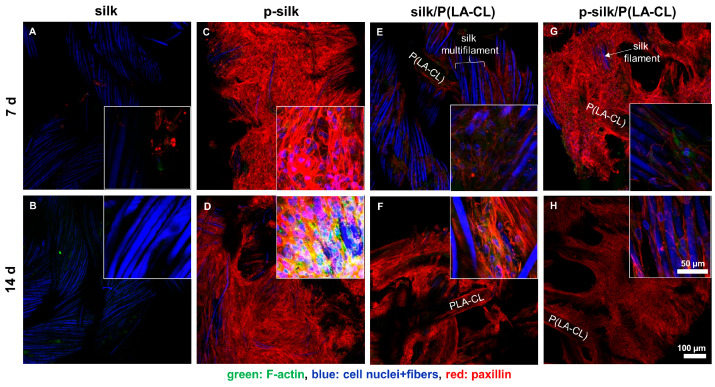
(**A**–**H**) The seeded scaffold types were immunolabeled for paxillin (red) as a crucial component of the focal adhesions and F-actin (green), indicating intimate cell-material interaction. Cell nuclei and scaffold fibers were counterstained in blue using 4′,6-diamino-2-phenylindole (DAPI). 100× magnification, scale bar: 100 µm. Insets: cells of another donor at 400× magnification. Scale bar: 50 µm. Except for the untreated silk scaffolds, LCL fibroblasts are mostly aligned along the fiber direction. (**A**,**B**) untreated silk, (**C**,**D**) purified (p-)silk, (**E**,**F**) untreated silk/P(LA-CL), (**G**,**H**) purified silk/P(LA-CL). P(LA-CL): poly-L-lactic acid-co-caprolactone. (**A**,**C**,**E**,**G**) 7 d. (**B**,**D**,**F**,**H**) 14 d.

Immunolabeling was performed to assess the main extracellular matrix ECM component (collagen type 1) and cytoskeletal paxillin, an adaptor protein of focal adhesion sites, hence, an essential component of cell-material adhesion. LCL fibroblasts seeded on untreated silk showed only focal expression of paxillin (Figure 9A,B), but after the integration of P(LA-CL) fibers into the scaffold (Figure 9E,F), a higher expression compared to the that on scaffolds consisting only of untreated silk became evident. The highest paxillin expression was seen in purified silk (Figure 9C,D) and purified silk/P(LA-CL) (Figure 9G,H) scaffold types, indicating high cell adhesions to the silk fibers. The scaffolds were also stained for F-actin. In contrast to the cover slips colonized with LCL fibroblasts and exposed to scaffold extracts, F-actin stress fibers could barely be seen at low magnification, and F-actin expression was generally weak. The highest expression of F-actin was observed in the purified silk scaffolds at 14 days (Figure 9D).

**Figure 10:**
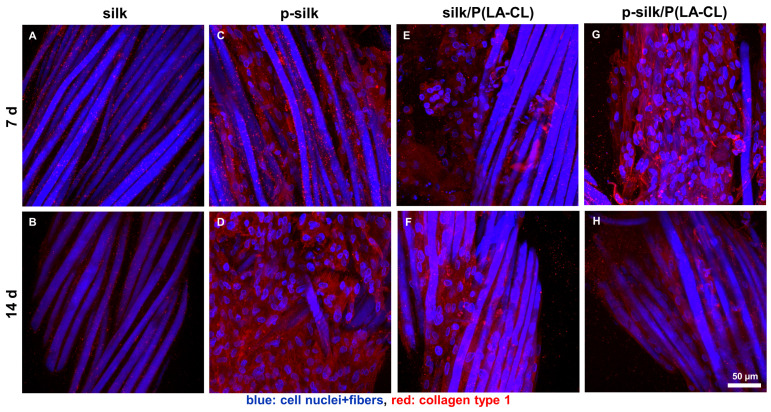
(**A**–**H**) Scaffolds were immunolabeled for collagen type 1 (red). The main ECM component of the ACL, collagen type 1, is detectable in all scaffold types, except for the untreated silk scaffolds. Cell nuclei and scaffold fibers were counterstained in blue using 4′,6-diamino-2-phenylindole (DAPI). Scale bar: 50 µm, 400× magnification. (**A**,**B**) untreated silk, (**C**,**D**) purified (p-)silk, (**E**,**F**) untreated silk/P(LA-CL), (**G**,**H**) purified silk/P(LA-CL). P(LA-CL): poly-L-lactic acid-co-caprolactone. (**A**,**C**,**E**,**G**) 7 d. (**B**,**D**,**F**,**H**) 14 d.

Similarly, scaffolds were also immunolabeled for collagen type 1 (Figure 10). Collagen type 1 is the major component of the ECM in ligament fibroblasts. Cells on untreated silk scaffolds exhibited only weak collagen deposits or intracellular expression (Figure 10A,B). Amongst the other scaffold types, purified silk mediated the highest expression of collagen type 1 in LCL fibroblasts (Figure 10C,D).

### 2.5. Ultramorphology of ACL Fibroblasts on Scaffold Variants Depicted by Scanning Electron Microscopy (SEM)

**Figure 11:**
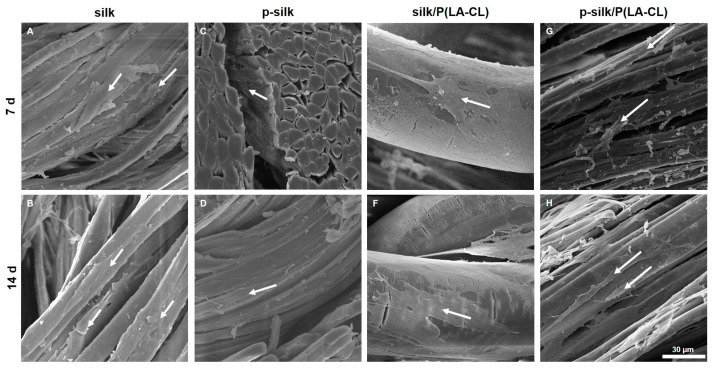
(**A**–**H**) The seeded scaffold types were investigated by SEM. Scale bar: 30 µm, 1000× *g* magnification. LCL fibroblasts (white arrows) are mostly aligned following the fiber direction. (**A**,**B**) untreated silk, (**C**,**D**) purified (p-)silk, (**E**,**F**) untreated silk/P(LA-CL), (**G**,**H**) purified silk/P(LA-CL). P(LA-CL): poly-L-lactic acid-co-caprolactone. (**A**,**C**,**E**,**G**) 7 d. (**B**,**D**,**F**,**H**) 14 d.

SEM revealed tightly packed bundles composed of multiple silk filaments. All scaffold variants revealed cell colonization. ACL fibroblasts were closely attached to the fibers and followed fiber direction. Cells had very elongated ligamentocyte-like shapes on silk multifilaments. In contrast, on the P(LA-CL), they had a broad and more flattened phenotype. The fibers remained stable and had a smooth and homogenous surface, which did not change during the entire observation period of 14 days (Figure 11).

### 2.6. DNA and Sulfated Glycosaminoglycan Content per Cell During Cultivation Time

**Figure 12:**
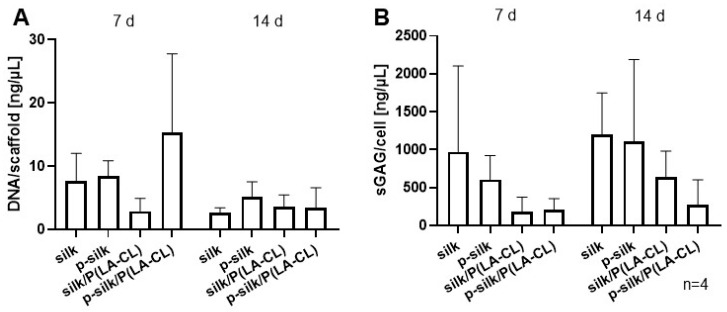
(**A**,**B**) Analysis of DNA (**A**) and sulfated glycosaminoglycan (sGAG, (**B**)) contents (day 7 and 14). sGAG content per cell based on the DNA content was determined in four independent experiments (*n* = 4) with cells from four different donors. P(LA-CL): poly-L-lactic acid-co-caprolactone; p-silk: purified silk.

The results of CyQuant assay are shown in Figure 12A. On day 7, the purified silk /P(LA-CL) scaffold type had the highest DNA content per scaffold, followed by purified silk, untreated silk, and untreated silk/P(LA-CL) scaffolds, respectively. On day 14, a decline in DNA content was observed in all the scaffold types, with purified silk having the highest DNA content. Sulfated glycosaminoglycans (sGAG) are the polysaccharide components of the ECM that help in hydration of the ECM. sGAG content per cell is shown in Figure 12B. On day 7, the untreated silk variant exhibited the highest sGAG expression. A thorough rise in sGAG content was observed in all the scaffolds on day 14, with untreated silk showing the highest expression, followed by p-silk group.

### 2.7. ACL Fibroblasts Express ACL-Related Genes on Scaffold Variants

**Figure 13:**
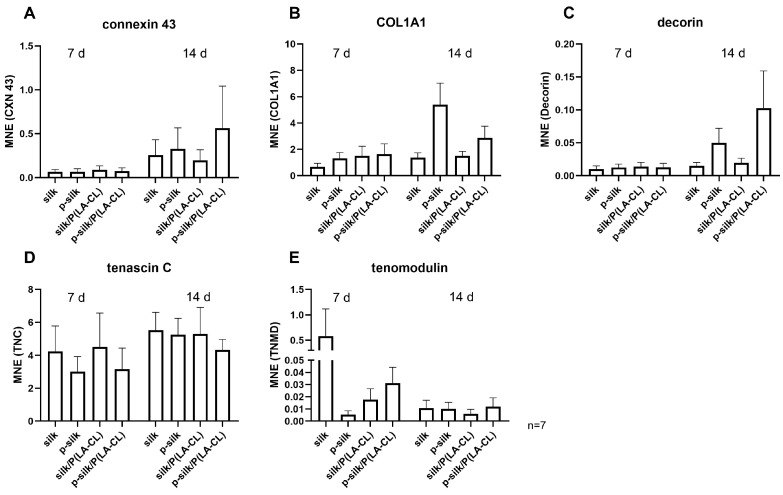
(**A**–**E**) Gene expression analysis (day 7 and 14) for connexin 43 (**A**), collagen type 1A1 chain ((**B**), COL1A1), decorin (**C**), tenascin C (**D**), and tenomodulin (**E**) in LCL fibroblasts cultured on different silk scaffolds. p-silk: purified silk; P(LA-CL): poly-L-lactic acid-co-caprolactone; MNE: mean normalized expression. Gene expression was normalized against the reference gene glyceraldehyde-3-phosphate dehydrogenase. Gene expression analysis was performed in seven independent experiments (*n* = 7) with cells from seven different donors. For better comparison, Y-axes was segmented in (**E**).

Gene expression analysis of connexin 43 was conducted for all the scaffold types on both day 7 and 14. Connexin 43 is a major component of the gap junctions that connect the cells and aid in cell–cell communication. Expression was noticed on both day 7 and 14, with purified silk/P(LA-CL) showing the highest expression on day 14 (Figure 13A). The comparison of day 7 and 14 revealed an overall increase in all scaffold groups on day 14.

Collagen type 1, one of the main ECM components, showed a comparable expression in all the scaffold groups on day 7; however, the expression was the highest in purified silk scaffolds on day 14 (Figure 13B). Like collagen type 1, decorin followed a similar trend in expression on day 7 (Figure 13C), but the expression was the highest in purified silk/P(LA-CL) scaffolds, followed by the purified silk variant at 14 days. Relatively high expression of tenascin C was noticed in all the scaffold groups on both day 7 and 14, with almost equal expression in all the groups on day 14 (Figure 13D). Tenomodulin, a marker for tenocytes and ligamentocytes, had low expression on day 7 (Figure 13E) except for untreated silk, but it was followed by an overall decrease in all the scaffold groups on day 14.

## 3. Discussion

A questionnaire by Rathbone et al. asking surgeons whether they would be open to use tissue-engineered ACL revealed that 86% of the surgeons gave a positive response, provided that the material offers biological and mechanical resilience [3]. Various techniques such as braiding and knitting have been previously employed to bioengineer ACL scaffolds [17,18,19,20]. Silk scaffolds, in combination with tenocytes, have previously been used for tendon repair procedures, such as for rotator cuffs [21]. Although silk has already been used for ACL tissue engineering as knitted scaffolds in a rabbit model [22], braided cord in a pig model [11], and as a rope-like scaffold in a sheep model [8], the use of embroidered silk scaffolds still remains unexplored. Nevertheless, embroidered PLA/P(LA-CL) scaffolds have been investigated thoroughly for ACL bioengineering in vitro [23] and in vivo using a nude mice model [24]. The advantage of silk for ACL bioengineering is its high biomechanical strength and durability [12]. This was proven in the present study, showing that the scaffolds had high stability, resembling the parameters required for ACL [12]. By selecting an appropriate embroidery pattern, embroidering enables the design of suitable topology and adapted biomechanics [25]; hence, the present study explores the use of embroidered silk scaffolds for ACL bioengineering to evaluate some important aspects of cell response such as cell survival and expression profiles for tendon related ECM components. The silk fibers used in this study comprised multifilaments with a diameter of around 189 µm. The fiber diameter can be influenced by the degumming process [26]. Degumming led to decreased diameters since sericin makes up around 20% of silk weight [27,28]. The single fibers of untreated and purified silk showed only a low range of variation in fiber diameters. However, this significant difference in diameters of silk single fibers (10.6 in untreated versus 12.4 µm in purified) could also be caused by the local source of silk (e.g., race of *B. mori*) since it is a natural material [29]. The overall fiber diameter is in agreement with other studies [30]. The silk single fiber diameters are within the range reported in the literature [12,31,32]. The fiber surface appeared smooth and unchanged, irrespective of the degumming process (untreated versus purified) and culture time (7 and 14 days). Altogether, these fiber dimensions were suitable for the embroidering process. The P(LA-CL) fiber monofilaments are substantially larger in diameter (≈90 µm) than the silk single-fibers and have been used in previous studies [4,24].

It is essential to assess the cytotoxicity of the biomaterial used for grafting, since it is foreign to the host cells and could possibly elicit an immune or adverse cell response [15,33]. Moreover, manufacturing and embroidering procedures may introduce toxic contaminants, for example, from water contaminated with lipopolysaccharides, toxic chemicals, or unfavorable pH; hence, we performed thorough cytotoxicity testing of the disinfected scaffolds. One has also to consider that the degumming process introduces chemicals or pH changes, and the embroidering under unsterile conditions might also affect cytocompatibility. As per the cytotoxicity assay results, the scaffold material did not prove to be harmful to either the LCL or L929 fibroblasts, since the measured values remained over the cytotoxicity thresholds. L929 fibroblasts are usually used for cytotoxicity testing following DIN ISO 10993-5:2009, since this cell line is a sensitive indicator of cytotoxicity [34]. LCL fibroblasts were included for comparison with the assumption of some cell-specificity, since this cell type was used in all other experiments of the study. Consistent with this assumption, LCL fibroblasts showed a lower cytotoxic response than L929 fibroblasts, with a significant difference in the purified P(LA-CL) scaffold group. The difference between both cell types has already been visualized previously by testing extracts of functionalized synthetic scaffold materials in a similar manner [34]. In agreement with the MTS cytotoxicity assay, which is based on enzymatic activity, no difference in cell viability was detected by the life/death assay. In addition to direct cytotoxicity, silk extracts might also exert other effects; for this reason, collagen synthesis and cell morphology was analyzed. LCL fibroblasts exposed to extracts showed neither morphological differences nor significant impairments in collagen synthesis compared to the negative control. The significant difference between the effects of purified silk extract and DMSO may be due to dying cells upregulating type I collagen expression. Interestingly, the F-actin fiber expression and stress fiber arrangement was significantly downregulated in cells treated with scaffold extracts compared to the control. These observations favored the continuation of our experiment into the next phases by seeding LCL fibroblasts on the scaffolds. In the viability assay, the highest and lowest cell attachment of living LCL fibroblasts was offered by purified silk and untreated silk, respectively. Notably, addition of P(LA-CL) to untreated silk improved cell attachment. As per the viability assay and immunolabelling results, purified silk offered highest adherence of living cells and collagen reactivity, respectively, while untreated silk showed the inferior results with the lowest colonization by vital LCL fibroblasts and ECM formation. The addition of synthetic fibers such as P(LA-CL) to untreated silk increased the adherence of living cells—a finding which is also in concurrence with the findings in the current literature, e.g., it has been reported for the combination of PLLA with silk fibroin [35]. Low F-actin expression under 3D scaffold conditions was also observed in another type of scaffolds when lacking mechanical challenges [36]. Obviously, some down-regulation appeared already after 48 h treatment with the silk scaffold extracts; hence, the overall low expression of F-actin stress fibers could be explained by lack of mechanical stretching of the scaffolds, since stretch increases the expression of F-actin stress fibers [37,38]. Nevertheless, F-actin could be shown on PLA/P(LA-CL) scaffold 3D culture, even in the absence of cyclic tension [23] suggesting that the presence of silk could somehow influence its expression in the present study.

The F-actin-containing cytoskeleton is linked to focal adhesion sites, which represent the dynamic and mechanical connections between the cell and their ECM or even scaffold environment. Paxillin is a crucial component of these focal adhesion sites. The latter mediate outside-in and subsequent inside-out signaling of the cell [39]. Paxillin expression could be visualized on cells growing on all scaffold variants tested, but was more abundantly expressed on purified silk and the combinations with P(LA-CL) fibers, underlining the intimate cell/material interaction. Synthesis of collagen type 1 by LCL fibroblasts could be visualized in all scaffold types. The DNA content did not show significant differences, but decreased after 14 d, whereas the gene expression analysis showed an increase in ECM component expression after 14 d. This could suggest cell differentiation rather than proliferation over time. The DNA content might be influenced by cutting the scaffolds after 7 days in two halves.

The next step was to assess whether the cells expressed the gene encoding the principal component of intercellular junctions on the scaffold variants. This was confirmed by the expression of connexin 43, which is a major component of the gap junctions [40]. Gene expression analysis showed, on day 14 of culture, that connexin 43 was expressed higher in p-silk scaffolds without or with P(LA-CL) fibers. Collagen type 1 and decorin showed a similar trend like connexin 43. Tenascin C is known as a fibroblast marker, and it is a mechanically regulated important structural glycoprotein in the human ACL; hence, it is also expressed by human ACL ligamentocytes [41]. Accordingly, it was transcribed in LCL fibroblasts grown on all scaffold variants, particularly those with integrated P(LA-CL) fibers, with no major differences. The expression of tenomodulin, a marker of ligamentocytes and tenocytes [42], confirmed the growth and ligament identity of LCL fibroblasts in the scaffolds. Despite highly expressed on untreated silk on day 7, no major differences could be detected between the investigated groups on day 14. One explanation for the decrease in tenomodulin expression could be that the mechanical stimulus was too weak, as tenomodulin is highly mechanosensitive [43]. The substrate, cell number, and 2D/3D culture conditions all influence tenomodulin expression [44].

Our study has some limitations, such as a short scaffold incubation period of only 14 days, the use of only animal-derived cells, a lack of external stretching to assess the mechanical strength, and the reported data only depicts the in vitro results. However, it presents a starting point for future studies for gaining a better understanding of silk fibroin as ACL substitute.

The present study underscores the critical interplay between material composition, scaffold architecture, mechanical performance, and cellular response in the design of embroidered scaffolds for ACL tissue engineering. Our comparative analysis of untreated silk, purified silk (p-silk), and their respective hybrid constructs with P(LA-CL) reveals that subtle differences in thread processing and scaffold configuration directly translate into distinct in vitro outcomes.

The embroidery approach enabled the fabrication of scaffolds with a reproducible, hierarchical arrangement that mimics ligamentous architecture. Structural analysis showed that untreated silk scaffolds were highly compact, with a porosity of ≈38%, whereas purified silk exhibited an intermediate porosity of ≈46%. Incorporation of P(LA-CL) monofilaments substantially increased scaffold porosity to ≈70%, thereby providing greater space for cell penetration and nutrient diffusion, but at the expense of mechanical competence. Purified silk scaffolds achieved the most favorable balance, combining sufficient mechanical stability with enhanced biological performance, as reflected by robust cellular colonization and extracellular matrix deposition. These results align with previous reports demonstrating that larger pores facilitate nutrient transport and cell infiltration, but can compromise the mechanical integrity essential for ligament function [40,41,42]. Thus, scaffold design must carefully balance porosity to optimize both biological permissiveness and load-bearing capacity, a principle broadly applicable across diverse biomaterial systems [45,46,47]. Uniaxial tensile testing confirmed that scaffold load-bearing capacity largely reflects the intrinsic properties of the constituent threads, with scaffolds composed solely of purified silk exhibiting the highest maximum forces (≈684 N). In contrast, elongation at maximum force did not directly correspond to the intrinsic extensibility of the threads. For instance, although the synthetic monofilament P(LA-CL) exhibits a substantially higher intrinsic elongation (≈95%), scaffolds containing P(LA-CL) as the lower thread showed markedly lower elongation (silk/P(LA-CL) ≈ 28%, p-silk/P(LA-CL) ≈ 32%) compared to non-hybrid silk scaffolds (silk ≈ 65%, p-silk ≈ 51%). This indicates that the scaffold’s overall extensibility is not only determined by the mechanical properties of the individual threads, but also by their interactions within the embroidered architecture. Specifically, the path and tension of the lower thread, intersections between threads, and stitch geometry create points where deformation of the silk fibers is mechanically restricted, leading to lower global elongation than would be expected from the properties of the single threads alone.

Embroidered scaffold architecture critically influences both cellular responses and mechanical performance. While ultimate tensile force reflects the intrinsic properties of the threads, scaffold extensibility is constrained by thread interactions, stitch geometry, and bobbin positioning. These insights provide a framework for designing embroidered silk-based scaffolds that meet the functional demands of ACL tissue engineering. The limitations of this study include the fact that no long-term cultures (4–8 weeks in vitro) were performed to investigate degradation, mechanical stability, the possible formation of fibrous tissue, and possible calcification. These are topics that will also be investigated in the future using human cruciate ligament fibroblasts.

## 4. Materials and Methods

### 4.1. Fiber Materials

In this study, two types of silk threads were employed: an untreated variant containing sericin (silk, linear density 206 dtex) and a degummed variant (p-silk, Shantung silk yarn, 100/120, 2-ply; linear density ≈ 166–200 dtex). Degumming was performed using a patented ultrasound-assisted process (DE102021118652A1). Both silk variants were supplied by Seidenkokon Native Silk GmbH (Nebelschütz, Germany)

The resorbable synthetic monofilament P(LA-CL), a poly(L- lactic acid-co-ε-caprolactone) (USP 6-0; linear density 80 dtex) was obtained from Gunze Ltd. (Tokyo, Japan).

### 4.2. Scaffold Manufacture Using Machine Embroidery

Scaffolds were produced by machine embroidery (TLMX-901, Tajima Industries, Nagoya, Japan) on a water-soluble nonwoven polyvinyl alcohol (PVA) fabric (Freudenberg Einlagestoffe KG, Weinheim, Germany) serving as a temporary support. Both silk thread types (Section 4.1) were used, either alone or combined with the synthetic monofilament P(LA-CL) applied as the lower thread. A zigzag embroidery pattern (stitch length 1.8 mm, stitch angle 15°, duplication shift 0.2 mm) was employed with upper and lower threads, and three embroidered plies were stacked using a silk locking thread, as previously described [36]. The resulting scaffolds replicated the dimensions of the rabbit ACL (12 × 4 × 2–3 mm).

After embroidery, the PVA support was removed by rinsing three times for 30 min in distilled water (A. dist.) on a compact shaker (KS 15 A, Edmund Bühler GmbH, Bodelshausen, Germany). The porous scaffolds were subsequently dried at room temperature (RT).

For sterilization, constructs were treated with 70% ethanol (Carl Roth GmbH + Co. KG, Karlsruhe, Germany) in a vacuum chamber for 24 h, followed by thorough rinsing with PBS (Pan-Biotech GmbH, Aidenbach, Germany). Accordingly, four scaffold groups were fabricated, as shown in the Table 1 below.

### 4.3. Fiber and Scaffold Characterization

#### 4.3.1. Light Microscopy

Microscopic imaging was performed on an inverted light microscope (Carl Zeiss, ZEN Core software, version: 2.7.80.0006) equipped with an Axiocam 305 color digital camera. Images were acquired using a 2.5× objective lens in a tile scanning mode (3 × 3 tiles, meander pattern, 10% overlap) at a fixed focal plane. Acquisition parameters included an exposure time of 10 ms with manual control (auto-exposure disabled) and a 12-bit color depth (Bgr48). Illumination was provided by an LED light source, with white balance and gamma correction (γ = 0.45) applied during acquisition.

#### 4.3.2. Scanning Electron Microscopy

Surface morphology of unseeded scaffolds was analyzed by scanning electron microscopy (SEM, ULTRA PLUS, Carl Zeiss Microscopy GmbH, Jena, Germany) equipped with an SE2 detector. Samples were sputter-coated with a 3 nm platinum layer to prevent charging. Micrographs were acquired at an accelerating voltage of 2.0 kV, a working distance of 8.7 mm, a 30 µm aperture, and 100× magnification. SEM was also performed after immunolabeling (see Section 4.8).

Scaffolds seeded with cells were also used for SEM using the following protocol: Scaffolds were fixed with 2.5% glutaraldehyde (Carl Roth GmbH) in PBS without Ca^2+^ and Mg^2+^ (Pan-Biotech GmbH) overnight at 4 °C. Subsequently, the scaffolds were washed in PBS four times for 15 min, and secondary fixation was carried out using 1% osmium tetroxide for 2 h (OsO4, Carl Roth GmbH) at RT. The scaffolds were gently rinsed in PBS four times for 15 min and dehydrated in an ascending ethanol series (70%, 80%, 90%, and 96% ethanol, each incubation step lasted 30 min) and three times in 99.6% ethanol for 15 min each. These steps were followed by drying with Hexamethyldisilazane (Carl Roth, 2 × 10 min at RT), mounting onto specimen stubs and sputtering (agar auto carbon coater, BalTEC COD 030, Agar scientific Ltd., Essex, UK) with a thin layer of carbon (≈13 nm). SEM images were taken using an ESEM XL30 (FEI Europe B.V., Eindhoven, The Netherlands) at an accelerating voltage of 15 kV.

#### 4.3.3. Micro-Computed Tomography (µCT)

High-resolution X-ray micro-computed tomography (µCT) was performed using a ProCon CT-ALPHA system (ProCon X-Ray GmbH, Sarstedt, Germany) using an x-ray energy of 80 keV and a tube target current of 200 μA. Projections were acquired with a detector pixel size of 99 µm and reconstructed to a voxel size of 7.5 µm. The source-to-object and source-to-detector distances were 3.8 cm and 50 cm, respectively. Reconstructions were carried out using the software X-AID (Version 2022.12.3, MITOS GmbH, Hamburg, Germany) using the Feldkamp–Davis–Kress (FDK) algorithm and a Hamming filter. A Gaussian filter (σ = 0.5) and partial ring-artifact suppression (size = 5) were applied.

#### 4.3.4. Determination of the Porosity of Textile Scaffolds

Porosity of the scaffold samples was determined from high-resolution µCT data using the software VG StudioMAX (Version 3.5, VolumeGraphics GmbH, Heidelberg, Germany). A three-dimensional region of interest (ROI) encompassing the entire sample was defined. Surface determination was applied to segment the scaffold material from air, providing the material volume. The volume of the pores was calculated as the difference between the total ROI volume and the material volume. Porosity was expressed as the ratio of the pore volume to the total ROI volume, providing a quantitative measure of the void space within the scaffold structure.

#### 4.3.5. Uniaxial Tensile Test

Mechanical properties of the fiber materials and scaffold specimens were evaluated by uniaxial tensile testing. Individual fibers were mounted without pre-tension in pneumatic rubber-coated clamps (20 N) on a Zwick Roell 0.5 machine equipped with a 100 N load cell (gauge length 50 mm, test speed 50 mm/min). Scaffold specimens were tested on a Zwick/Roell UPM 2.5 machine (ZwickRoell GmbH & Co. KG, Ulm, Germany) using a 1000 N load cell and pneumatic metal clamps (Zwick 8195.05, broad serrated, 4 bar) over a 10 mm gauge length with a 0.1 N pre-load and a test speed of 10 mm/s, in accordance with DIN EN ISO 13934-1. Force data were recorded with TestXpert software (https://www.zwickroell.com/accessories/testxpert-testing-software/ accessed on 25 October 2022), and the maximum force (F_max [N]) and strain at maximum force (ε_Fmax [%]) were reported for each specimen. Between five and ten fibers or five scaffold specimens were tested per material under controlled conditions (23 °C, 50% relative humidity).

### 4.4. Lapine Cruciate Ligament Fibroblast Isolation

Cruciate ligaments of twelve healthy New Zealand rabbits (approximately 14 months old), derived from an abattoir, were used to isolate LCL fibroblasts. After explanting the LCLs, they were sliced into 2 mm^2^ pieces and placed in a T25 culture flask (Sarstedt AG & Co. KG, Nürnbrecht, Germany) with growth medium (Dulbecco’s Modified Eagle’s Medium (DMEM)/Ham’s F12 medium (Pan-Biotech GmbH) supplemented with 10% fetal bovine serum (FBS, Pan-Biotech GmbH), 1% penicillin/streptomycin solution (Pan-Biotech GmbH), 25 µg/mL ascorbic acid (Sigma-Aldrich, Munich, Germany), 2.5 µg/mL amphotericin B (Pan-Biotech GmbH), and 1% MEM amino acid solution (Pan-Biotech GmbH) for several weeks. Growth medium was changed from every second to third day.

After 7–10 days, the emigrating LCL fibroblasts were first rinsed with PBS and then harvested with 0.05% trypsin/0.02% EDTA (Pan-Biotech GmbH) and collected in growth medium.

### 4.5. Cytotoxicity Testing

For the evaluation of cytotoxicity, L929 mouse subcutaneous fibroblasts and LCL fibroblasts were used. The cytotoxicity testing procedure was carried out according to the respective ISO standard to analyze any potential cytotoxic effects. Sterile extracts of all scaffold types were obtained after incubating them for 48 h in growth medium (one scaffold/mL growth medium). L929 and LCL fibroblasts were seeded at 10 × 10^4^ cells/well in 96-well culture plates for 24 h at 37 °C and 5% CO_2_. Biological evaluation of cytotoxicity was performed according to the international standard DIN EN ISO 10993-5 2009-10 norm. In addition, 25 × 10^4^ cells were seeded on cover slips in a 48-well plate. After 24 h cell adhesion time in growth medium, the supernatant was completely exchanged by scaffold extracts (volume 100 µL extract/well, triplicates) or triplicates of control solutions for 24 h at 37 °C and 5% CO_2_. Moreover, 100 µL 10% dimethyl sulfoxide (DMSO, Carl Roth GmbH and Ko.KG, Karlsruhe, Germany) diluted in the growth medium was used as the positive control, and pure growth medium served as the negative control. After incubating the cells for 24 h, all supernatants were removed and a mixture of 80 µL growth medium and 20 µL [3-(4,5-dimethylthiazol-2-yl)-5-(3-carboxymethoxyphenyl)-2-(4-sulfophenyl)-2H-tetrazolium, inner salt; MTS] solution (CellTiter 96^®^ Aqueous One Solution Cell Proliferation Assay, Promega GmbH, Walldorf, Germany) was added to each well. Cells were exposed to the reagent for 2 h before absorbance was measured at a wavelength of 490 nm and a reference wavelength of 630 nm (Infinite M200, Tecan Austria GmbH, Grödig, Austria).

### 4.6. Scaffold Seeding

The 4 scaffold types were seeded with LCL fibroblasts, with a target of at least 2 million cells/scaffold. They were incubated in 10 mL culture medium in rotator tubes on a rotatory device (Bartelt GmbH, Graz, Austria) with 36 rounds per min (rpm) at 37 °C. Scaffolds were divided into 2 halves at day 7. One half was put back in the growth medium and incubator, and the other half, sectioned in two quarters, was used for the life/death assay and immunolabeling. At day 14, the remaining half was subjected to the same assays as mentioned above. For gene expression analysis, a whole scaffold was used for each time point of the analysis (7 and 14 d).

### 4.7. Life/Death Assay

In total, 1 mL of PBS was mixed with 5 µL of fluorescein diacetate (FDA, Sigma-Aldrich) stock solution (5 mg/mL in acetone) and 1 µL of propidium iodide (PI, Thermo Fisher Scientific, Darmstadt, Germany) stock solution (2 mg/mL in PBS). The first dye labeled the living cells green, whereas the second stain labeled the dead cells red. In total, 50 µL of the staining solution was placed on a thin glass slide (0.16–0.19 mm, R. Langenbrinck, Emmendingen, Germany), the sample (LCL fibroblast colonized scaffold segment or cover slip with cells) was immersed and another 50 µL drop was added to the sample. After a 5 min incubation, the sample was ready to be observed using a Leica CLSM with Leica LASX software (Leica TCS SPEII and DMi8, software version 3.5.7.23225, Leica Microsystems, Wetzlar, Germany) to take pictures. The results of the life/death assay of cover slips were used to estimate the surface covered by living cells using ImageJ1.48 v software.

### 4.8. Immunofluorescence Labeling

Small sections of scaffolds were fixed in 4% paraformaldehyde (MORPHISTO GmbH, Frankfurt am Main, Germany) at RT. Glass cover slips seeded with LCL fibroblasts and exposed to extracts were also stained for phalloidin-Alexafluor(A)488 and a collagen type I antibody. For staining, fixed scaffold segments or cover slips were rinsed three-times with Tris buffered saline (TBS: 0.05 M Tris, 0.15 M NaCl, pH 7.6, Carl-Roth GmbH), and were later overlaid with blocking buffer (5% donkey serum [Chemicon, Temecula, CA, USA], 0.1% Triton X100 [Sigma-Aldrich] diluted in TBS) for 20 min at RT). Afterwards, the samples were incubated with primary antibodies (paxillin and collagen type 1; see Table 2) overnight at 4 °C. For this, 50 µL of a solution containing the antibodies for paxillin and collagen type 1 was added to each scaffold. On the next day, the samples were rinsed three times with TBS for 5 min each. Then, secondary antibody incubation was performed for 1 h at RT. The secondary antibody solution was composed, as depicted in Table 2, with a dilution ratio of 1:200 in blocking solution (see Table 2). To stain nuclei, 4′,6-diamino-2-phenylindole (DAPI, 10 µg/mL, Roche, Mannheim, Germany) was added to the suspension of the secondary antibodies. Phalloidin-Alexafluor (A)488 stain (1:100, Santa Cruz Biotechnologies, Inc., Dallas, TX, USA) was used to visualize the filamentous (F)-actin cytoskeleton. Afterwards, the samples were washed three times with TBS and covered with a cover slide (R. Langenbrinck) and fluoromount G (SouthernBiotech, Birmingham, AL, USA) before being examined by CLSM. Fiber dimensions were measured in three representative images of the scaffolds using DAPI stain and the measurement function of LAS software (Leica, software version 3.5.7.23225). After the immunofluorescence labeling, the scaffolds were fixed in 4% paraformaldehyde (PFA, Morphisto GmbH, Frankfurt am Main, Germany) and stored at 4 °C until further use for SEM.

### 4.9. Measurement of Total DNA and Sulfated Glycosaminoglycan Content

The CyQuant assay was used (Invitrogen, Carlsbad, CA, USA) to measure the DNA content of the scaffolds. Bovine thymus DNA served as a reference standard. First, all the scaffolds colonized with LCL fibroblasts were homogenized with a 7 mm stainless steel bead (RNase and DNase free, sterile, Qiagen, Hilden, Germany) by using TissueLyser LT (Qiagen, 50 Hz, 5 min, RT). In the next step, samples were subjected to incubation with proteinase K (PanReac, ApplyChem, Darmstadt, Germany) solution for digestion (0.5 mg/mL, Carl Roth GmbH and Ko.KG), dissolved in 50 mM Tris/HCl, 1 mM EDTA, 0.5% Tween20, pH 8.5) for 24 h at 56 °C under continuous shaking (36 rpm). Afterwards, samples were centrifuged for 30 min at 10,000 rpm at RT. In total, 10 µL sample and 150 µL Tris-EDTA (TE) buffer were mixed. Then, the triplicates of specimens (25 µL each) were transferred to a black 96-well plate with a flat bottom (Brand GmbH, Wertheim, Germany), and 25 µL dye solution (1x Hank‘s Balanced Salt Solution [HBSS, Pan-Biotech GmbH] + dye solution 1:250) was added. Plates were photoprotected and were incubated at 37 °C for 60 min. Fluorescence was measured at λ = 485 excitation and λ = 530 emission using a fluorometric plate reader (Infinite M200 Tecan reader). The cell content of the scaffolds was calculated based on the assumption that each cell contains approximately 7.7 pg DNA [48]. The dimethylmethylene blue (DMMB) assay was used to measure sGAG contents. The specimens were first diluted in phosphate-buffered ethylendiaminetetraacetic acid (EDTA) buffer (PBE, 100 mM Na_2_HPO_4_, and 5 mM EDTA, pH 8), and were later exposed to the DMMB staining solution (8.9 mM DMMB hydrochloride in 600 mg glycine, 467 mg NaCl and 200 mL A. dist.). Afterward, the absorption shift from λ = 525 nm to λ = 595 nm was immediately assessed (Tecan reader). sGAG content was calculated with chondroitin sulfate (Sigma-Aldrich) as a reference standard.

### 4.10. RNA Isolation

All of the scaffold types colonized with LCL fibroblasts were snap-frozen after day 7 and 14, and were homogenized in RLT-buffer (Qiagen) with the Tissue Lyser for 2 times for 3 min at 50 Hz. RNA was isolated using the RNeasy Mini kit according to the manufacturer’s protocol (Qiagen), which also included on-column DNAse treatment for genetic DNA removal. The purity and quantity of the RNA samples (260/280 nm absorbance ratio) was monitored using the Nanodrop ND-1000 spectrophotometer (Peqlab, Biotechnologie GmbH, Erlangen, Germany).

### 4.11. Gene Expression Analysis

cDNA synthesis was performed with 625 ng of total RNA and the reverse transcription kit (QuantiTect Reverse Transcription Kit, Qiagen) according to the supplier’s protocol. Quantitative real-time PCR (qRT-PCR) reactions were carried out using 25 ng cDNA and the TaqMan Gene Expression Assay (Life Technologie, Darmstadt, Germany) with primers for collagen type 1 (*COL1A1*), decorin (*DCN*), tenascin C (*TNC*), tenomodulin (*TNMD*), connexin 43 (*CXN43*), and the reference gene glyceraldehyde 3-phosphate dehydrogenase (*GAPDH*) (Table 3). Furthermore, the real-time PCR detector StepOnePlus (Applied Bioscience [ABI], Foster City, CA, USA) thermocycler with the program StepOnePlus software 2.3 (ABI) was used for qRT-PCR. To assess the relative expression of the gene of interest by the cells. The expression was normalized to the GAPDH expression, and expression was calculated for each sample using the ∆CT method described by Schefe and colleagues [49].

### 4.12. Statistical Analysis

All values (CyQuant Assay and DMMB Assay) were expressed as the mean with the standard deviation (SD) and relative gene expression was expressed as normalized mean expressions with the standard error of mean using GraphPad Prism 8 (GraphPad Software Inc., San Diego, CA, USA). The ROUT method of identifying outliers (Q = 1%) was used before testing the normal distribution of the results. Afterwards, ordinary one-way ANOVA and Tukey’s multiple comparison test were conducted to evaluate the significant differences between the groups. Statistical significance was set at a *p*-value ≤ 0.05 (*). The following *p* values were indicated: * *p* < 0.05, ** *p* < 0.01, *** *p* < 0.001, **** *p* < 0.0001.

At least three to seven independent experiments with cells derived from different donors were performed.

## 5. Conclusions

Altogether, the results indicate that non-cytotoxic scaffolds can be fabricated from both standard and purified silk fibers, alone or in combination with P(LA-CL) fibers. ACL fibroblasts were able to colonize the silk scaffolds, express ligament-related ECM components, and maintain gap junction communication via connexin 43, demonstrating the biological suitability of the constructs. Scaffold architecture critically influenced both cell behavior and mechanical performance: ultimate tensile force reflected the intrinsic properties of the threads, while scaffold extensibility was constrained by thread interactions and stitch geometry. Increased porosity enhanced cell infiltration and nutrient diffusion, but reduced mechanical competence, whereas intermediate porosity (≈46%, purified silk) achieved an optimal balance between biological activity and structural integrity. These findings provide a foundation for designing embroidered silk-based scaffolds that meet the functional requirements of ACL tissue engineering.

## Figures and Tables

**Table 1:** Designation and composition of the textile scaffolds.

Group Name	Material for Upper Thread	Material for Lower Thread
silk	silk, untreated	silk, untreated
p-silk	silk, purified	silk, purified
silk/P(LA-CL)	silk, untreated	P(LA-CL)
p-silk/P(LA-CL)	silk, purified	P(LA-CL)

**Table 2:** Antibodies and staining used for immunocytochemistry.

Target	Primary Antibody, Source	Dilution	Secondary Antibody, Source	Dilution
collagen type 1	goat anti human, SouthernBiotech, Birmingham, AL, USA	1:50	donkey-anti-goat cy3, Invitrogen, Carlsbad, CA, USA	1:200
paxillin	mouse-anti-human, BD Biosciences, Toronto, CA	1:40	donkey-anti-mouse cy3, Invitrogen, Carlsbad, CA, USA	1:200
phalloidin-Alexa488	Phalloidin-iFlour 488, Abcam, Cambridge, UK	1:100	-	
4′,6′-diamidino-2-phenylindol (DAPI)	Roche, Mannheim, Germany	1:100	-	

**Table 3:** Primer list for gene expression analysis.

Gene Symbol	Species	Gene Name	Amplicon Length	Assay ID
*COL1A1*	*O. cuniculus*	type 1 collagen, alpha 1 chain	70	Oc03396073_g1
*DCN*	*Homo sapiens*	decorin	77	Hs00370384_m1
*TNC*	*O. cuniculus*	tenascin C	61	Oc06726696_m1
*TNMD*	*O. cuniculus*	myodulin	146	Oc03399505_m1
*CXN43*	*O. cuniculus*	connexin 43	68	Oc03396056_g1
*GAPDH*	*O. cuniculus*	glyceraldehyde-3-phosphate dehydrogenase	82	Oc03823402_g1

O.: Oryctolagus.

## Data Availability

The data presented in this study are available on request from the corresponding author.

## Supplementary Materials

The following supporting information can be downloaded at https://www.mdpi.com/article/10.3390/ijms27010137/s1.

## Abbreviations

The following abbreviations are used in this manuscript:

3D	three-dimensional
ABI	applied bioscience
ACL	anterior cruciate ligament
B. mori	Bombyx mori
CLSM	confocal laser scanning microscope
COL1A1	collagen type 1 alpha chain
CXN43	Connexin 43
DAPI	4′,6-diamino-2-phenylindole
DCN	decorin
DMEM	Dulbecco’s Modified Eagle’s Medium
DMMB	dimethylmethylene blue
DMSO	dimethylsulfoxide
ECM	extracellular matrix
EDTA	ethylendiaminetetraacetic acid
FBS	fetal bovine serum
FDA	fluorescein diacetate
GAPDH	glyceraldehyde 3-phosphate dehydrogenase
HBSS	Hank‘s balanced salt solution
LCL	lapine cruciate ligament
MTS	3-(4,5-dimethylthiazol-2-yl)-5-(3-carboxymethoxyphenyl)-2-(4-sulfophenyl)-2H-tetrazolium inner salt
P(LA-CL)	PLA-co-caprolactone
PBS	phosphate-buffered saline
PI	propidium iodide
PLLA	poly-L-lactic acid
qRT-PCR	quantitative reverse transcription polymerase chain reaction
rpm	rounds per minutes
RT	room temperature
SD	standard deviation
SEM	Scanning electron microscopy
TBS	tris buffered saline
TE	Tris-EDTA
TNC	tenascin C
TNMD	tenomodulin
