# Supplementary figures and images for: Embroidered Silk Fibroin Scaffolds for ACL Tissue Engineering
Source: Int J Mol Sci. 2025 Dec 22;27(1):137. doi: 10.3390/ijms27010137 (PMC12786107; doi:10.3390/ijms27010137)

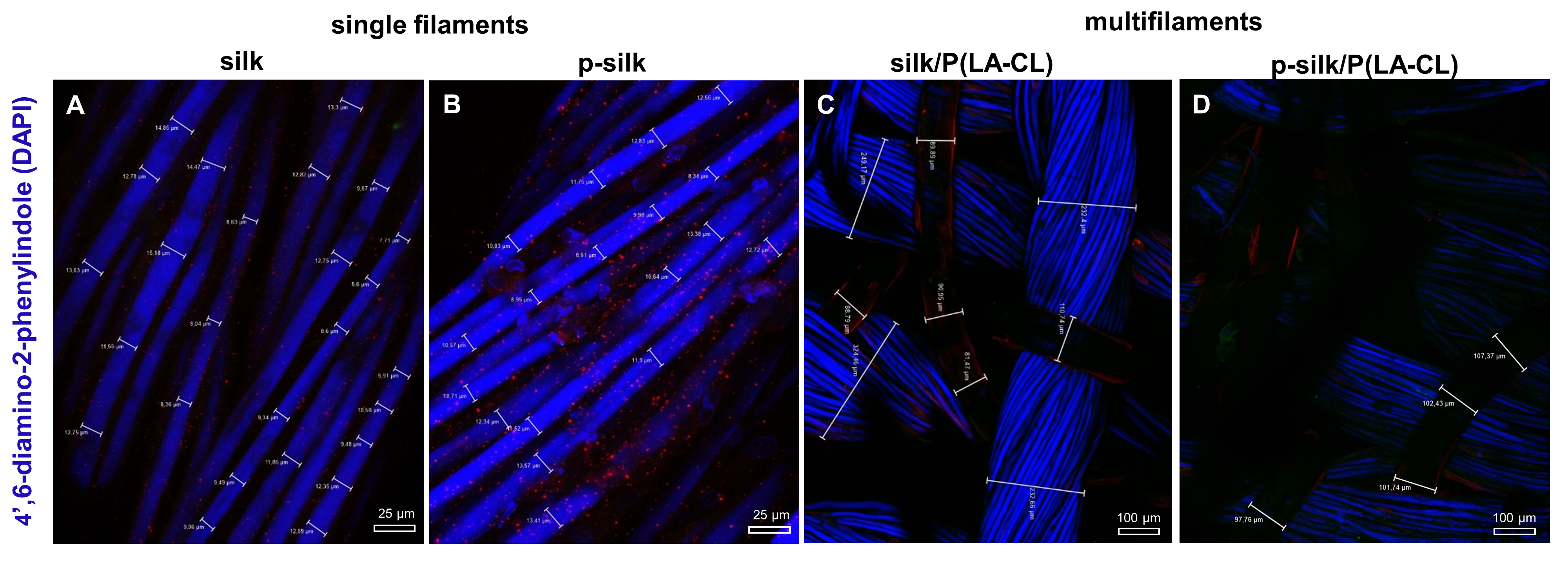

Supplement: Supplementary file 1 [file ijms-27-00137-s001.zip › ijms-3908823-supplementary.tif]
